# Evaluation of dysplasias associated with inflammatory bowel disease—a single-center, retrospective, 5-year experience

**DOI:** 10.3389/pore.2025.1612105

**Published:** 2025-04-15

**Authors:** Zsófia Balajthy, Panna Szaszák, Szintia Almási, Tamás Lantos, Anita Sejben

**Affiliations:** ^1^ Department of Pathology, Albert Szent-Györgyi Medical School, University of Szeged, Szeged, Hungary; ^2^ Department of Medical Physics and Informatics, Albert Szent-Györgyi Medical School, University of Szeged, Szeged, Hungary

**Keywords:** inflammatory bowel disease, non-conventional dysplasia, serrated NOS dysplasia, conventional dysplasia, colorectal carcinoma

## Abstract

**Introduction:**

Several novel morphological variants of inflammatory bowel disease (IBD)- associated dysplasias have been described in recent years. The objective of our study was to reevaluate some of our IBD-associated neoplasia cases and retrospectively identify the so-called non-conventional dysplasias (NCDs).

**Methods:**

We established a database of IBD patients registered between 2011 and 2015 at the Department of Pathology, University of Szeged. Patients with neoplastic samples were extracted into a separate database. Clinical and pathological characteristics were documented for each case. Histological slides were retrospectively reviewed, and cases were reclassified.

**Results:**

During the study period, 57 patients had neoplastic samples, and 47 patients were identified with conventional dysplasias (82.5%). A significant association was found between conventional dysplasias and dysplasia localization (*P = 0.004*), size (*P = 0.012*), endoscopic appearance (*P = 0.006*), grade (*P = 0.011*), macroscopic appearance of colorectal carcinoma (*P = 0.009*), and pT stage (*P = 0.01*). NCD was identified in 20 cases (35.1%). The most frequently observed subtype was serrated not otherwise specified (NOS) dysplasia (n = 6; 30%). Significant associations were detected between the development of NCD and several clinical-pathological features, including the occurrence (*P < 0.001*), localization (*P = 0.001*), size (*P = 0.002*), macroscopic appearance (*P = 0.01*), grade (*P = 0.005*), histological subtype (*P = 0.003*), pT (*P = 0.00*3) and pM stage (*P = 0.047*) of colorectal carcinoma, as well as microsatellite status (*P < 0.001*).

**Discussion:**

The identification of IBD-associated NCDs might play a crucial role in future clinical practice. Some authors suggest closer patient follow-up upon identification of these lesions and recommend random biopsy sampling in IBD patients to detect potentially occult lesions. Further studies involving larger national and international patient cohorts are warranted to gain a more comprehensive understanding of the clinical behavior of NCDs.

## Introduction

Inflammatory bowel disease (IBD) incorporates ulcerative colitis (UC), Crohn’s disease (CD), and indeterminate colitis. Due to the cumulative inflammation burden because of the long-standing and relapsing nature of IBDs, the altered microbiome, and individual immunological mechanisms, IBD predisposes patients to develop dysplasias earlier than in the control, non-IBD population [1, 2]. The mortality of IBD-associated colorectal carcinomas shows a decreasing tendency, due to the surveillance colonoscopies, and the advances in endoscopic resection and anti-inflammatory therapy [3, 4]. According to current guidelines, colorectal carcinoma screening should be initiated 8 years after IBD diagnosis [2, 3].

New IBD-associated dysplasias named non-conventional dysplasias (NCDs) have been introduced by Choi et al in [5], including hypermucinous, goblet cell-deficient (GCD), dysplasia with increased Paneth cell differentiation (DPD), crypt cell dysplasia (CCD), traditional serrated adenoma (TSA)-like, sessile serrated lesion (SSL)-like, and serrated lesion, not otherwise specified (NOS) [5]. Even though NCDs histologically usually reflect low-grade morphology, based on data mainly from the North American population, they have been associated with poorer prognosis, as they are more frequently associated with aneuploidy and advanced neoplasias. Some subtypes may appear flat during the endoscopic examination, making their detection challenging [5, 6]. These lesions have been observed more commonly in the same localization as colorectal carcinomas; therefore, they may serve as potential precursor lesions [7]. Furthermore, they have been associated with new subtypes of colorectal carcinoma, including tubuloglandular, GCD, and serrated variants [3, 8]. Given the limited information about these lesions, their clinical and histological identification remains difficult.

The aim of our study was the retrospective identification of IBD-associated NCDs within a consecutive single-center study, as well as the evaluation of clinicopathological parameters influencing the prognosis of NCDs.

## Materials and methods

All IBD patients diagnosed at the Department of Pathology, University of Szeged, Hungary, between 2011 and 2015 were identified based on ICD codes, and neoplastic samples from these patients were subsequently reviewed. During database construction, clinicopathological characteristics including the age, sex, type, duration, and localization of IBD were collected. The histological type, date of diagnosis, lesion size, and morphology observed during endoscopy were retrospectively documented in the case of dysplastic samples. In cases of invasive tumors, additional data including histological type, date of tumor diagnosis, grade, macroscopic morphology, localization, TNM stage, and the presence of vascular or lymphatic invasion. Progression-free survival (PFS) was solely defined in those cases who developed colorectal carcinoma during follow-up, while overall survival (OS) was defined in all cases.

Histological evaluation was utilized with an Olympus BX53F microscope. A diagnosis of NCD was established only when the observed lesion was present in at least 50% of the sample. If multiple types of NCDs were observed within a single slide, a dominant pattern was selected, based on the extent and size of the lesions for statistical analysis. This study does not address multiplex lesions; however, our research group plans to focus on this aspect in future investigations.

Statistical analyses were performed using Fisher’s exact test and chi-square test for discrete variables, as well as the Mann–Whitney U test and two-sample t-test for continuous variables. *P < 0.05* was considered statistically significant.

Our study was approved by the Medical Research Council (BM/28834-1/2024).

## Results

### General clinicopathologic characteristics

In our examined 5-year period, a total of 921 IBD patients were included, and 57 possessed dysplasia or carcinoma samples. The mean age of the patients was 63.7 years (median: 65; range: 33–94). A male predominance was observed (n = 34; 59.6%), and most patients were treated for ulcerative colitis (UC; n = 41; 71.9%). In half of the cases, IBD affected the left colon (n = 29; 50.9%), while pancolitis was reported in 17 cases (29.8%). The average duration of IBD prior to the diagnosis of neoplasia was 15 years (median: 14, range: 1–37).

### Clinicopathologic characteristics of IBD-associated conventional dysplasias

Altogether 47 patients were identified with conventional dysplasias. The average age of patients was 65.2 years (median: 66, range: 33–94), and male predominance was observed with a male-to-female ratio proved to be 31:16. Patients were mostly diagnosed with UC (n = 33; 70.2%), with left colon localization (n = 22; 46.8%), followed by pancolitis (n = 15; 31.9%). The average IBD duration until dysplasia diagnosis was 14.8 years (median: 14; range: 1–37). The dysplastic lesions were mainly identified in the left colon (n = 31; 65.9%). The average microscopic size of dysplasia was 0.66 cm (median: 0.5; range: 0.2–2.5), with mostly polypoid endoscopic morphology (n = 34; 72.3%).

**FIGURE 1:**
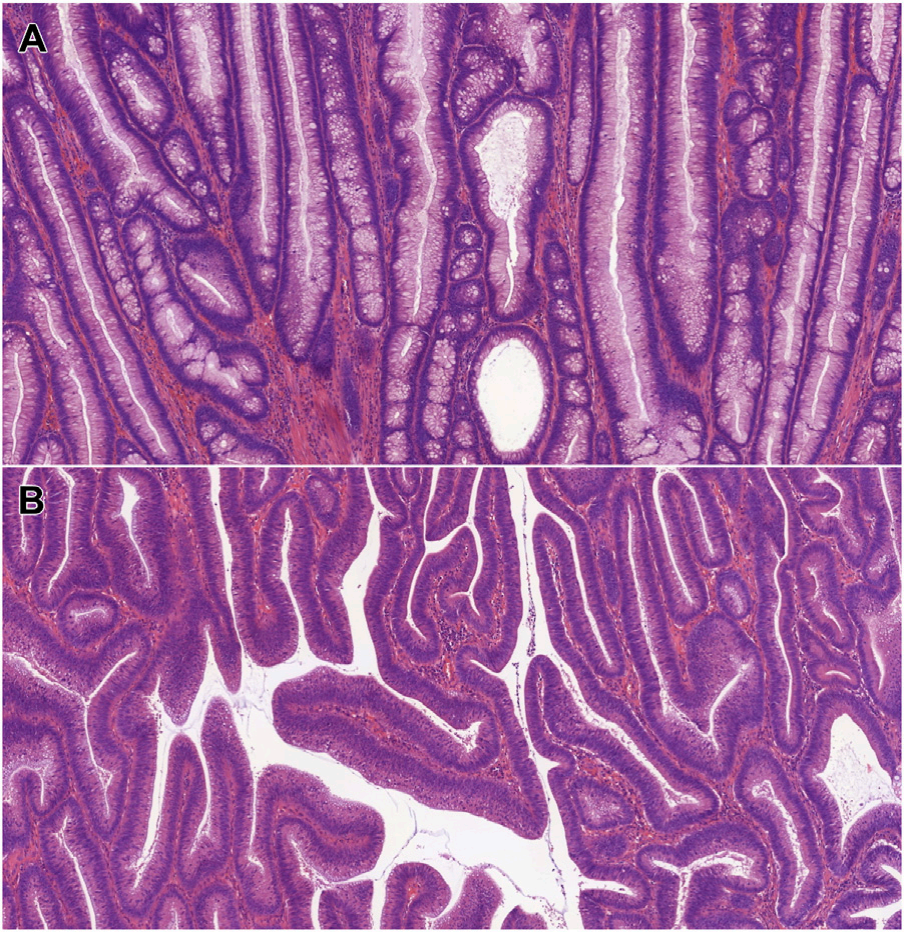
Microscopic features of IBD-associated, conventional dysplasias. **(A)**: IBD-associated tubular adenoma with low-grade dysplasia (HE, 5x). **(B)**: IBD-associated tubulovillous adenoma with low-grade dysplasia (HE, 5x). Abbreviations: HE - Hematoxylin and eosin, IBD - Inflammatory bowel disease.

The dominant histological pattern proved to be tubular adenoma in the majority of cases (n = 38; 80.8%), and tubulovillous adenoma was solely found in 9 cases (19.1%). Villous adenoma was not identified. Figure 1 represents conventional dysplasias found in our cohort. Forty cases (85.1%) were defined as low-grade. The clinicopathological features of the identified conventional dysplasias are summarized in Table 1.

**TABLE 1:** Clinicopathological characteristics of the identified conventional dysplasias.

	Tubular adenoma (n = 38)	Tubulovillous adenoma (n = 9)
Age at diagnosis (years)	Mean: 64 Median: 63 (33–94)	Mean: 70 Median: 67 (62–81)
Male-to-female ratio	26:12	5:4
Type of IBD (n)	UC: 28 CD: 10	UC: 5 CD: 4
Localization of IBD (n)	Left colon: 19 Right colon: 8 Pancolitis: 11	Left colon: 3 Right colon: 2 Pancolitis: 4
Duration of IBD (years)	Mean: 14.8 Median: 12 (1–37)	Mean: 17.9 Median: 19 (9–24)
Localization of dysplasia (n)	Left colon: 26 Right colon: 12	Left colon: 5 Right colon: 4
Grade of dysplasia (n)	Low-grade: 36 High-grade: 2	Low-grade: 9 High-grade: 0
Size of dysplasia (cm)	Mean: 0.7 Median: 0.5 (0.2–2.5)	Mean: 0.47 Median: 0.4 (0.3–1.1)
Endoscopic appearance of dysplasia (n)	Polypoid: 26 Flat: 12	Polypoid: 8 Flat: 1
Association with non-conventional dysplasia (n)	15	0
Association with adenocarcinoma (n)	11	1

Abbreviations: CD, Crohn’s disease; IBD, inflammatory bowel disease; UC, ulcerative colitis.

A significant association was found between conventional dysplasias and dysplasia localization (*P = 0.004*), size (*P = 0.012*), endoscopic appearance (*P = 0.006*), grade (*P = 0.011*), macroscopic appearance of colorectal carcinoma (*P = 0.009*), and pT stage (*P = 0.01*). There was no association found with the patient’s age (*P = 0.081*), gender (*P = 0.072*), subtype (*P = 0.708*), duration (*P = 0.817*) and extent of IBD (*P = 0.465*), development (*P = 0.058*), localization (*P = 0.064*), size (*P = 0.066*), grade (*P = 0.066*), multiplicity (*P = 0.619*), histological subtype (*P = 0.065*), pN (*P = 1.00*) and pM stage (*P = 0.208*) of colorectal carcinoma, the presence of lymphovascular invasion (*P = 1.00*), and microsatellite status (*P = 1.00)*.

### Evaluation of IBD-associated adenocarcinomas in patients with conventional dysplasia

Of the 47 patients with conventional dysplasia, 12 patients (25.5%) were diagnosed with colorectal adenocarcinoma during follow-up, that were mainly localized to the left colon (n = 8; 66.7%), with average size of 4 cm (median: 3.75; range: 1–8.4), and infiltrative morphology (n = 7; 58.3%). The majority were identified as low-grade (n = 10; 83.3%). In half of the cases (n = 6), multiple colorectal carcinomas developed. Histologically, 7 cases (58.3%) were characterized as conventional adenocarcinoma, followed by mucinous (n = 3; 25%), signet ring cell (n = 1; 8.3%), and GCD (n = 1; 8.3%) patterns. Altogether 8 cases (66.7%) were found in T3 or T4 stage. Lymphovascular invasion and distant metastases were both identified in 3-3 cases (25%), respectively. Microsatellite instability was identified in a single case (n = 1; 8.3%).

### Clinicopathologic characteristics of IBD-associated NCD

Among the 57 IBD patients with neoplastic samples, NCD was observed in 20 cases (35.1%). The mean age of these patients was 64.8 years (median: 66, range: 46–83), with a male-to-female ratio of 12:8. Most patients with NCD were diagnosed with UC (n = 14; 70%), and the IBD was predominantly localized to the left colon (n = 11; 55%). The average time from IBD diagnosis to the detection of dysplasia was also 15 years (median: 15, range: 4–28). Most dysplastic lesions were localized to the left colon (n = 17; 85%), with average size of 0.59 cm (median: 0.45; range: 0.2–2.5), and mostly flat endoscopic morphology (n = 12; 60%). The predominance of them were histologically evaluated as low-grade (n = 15; 75%).

**FIGURE 2:**
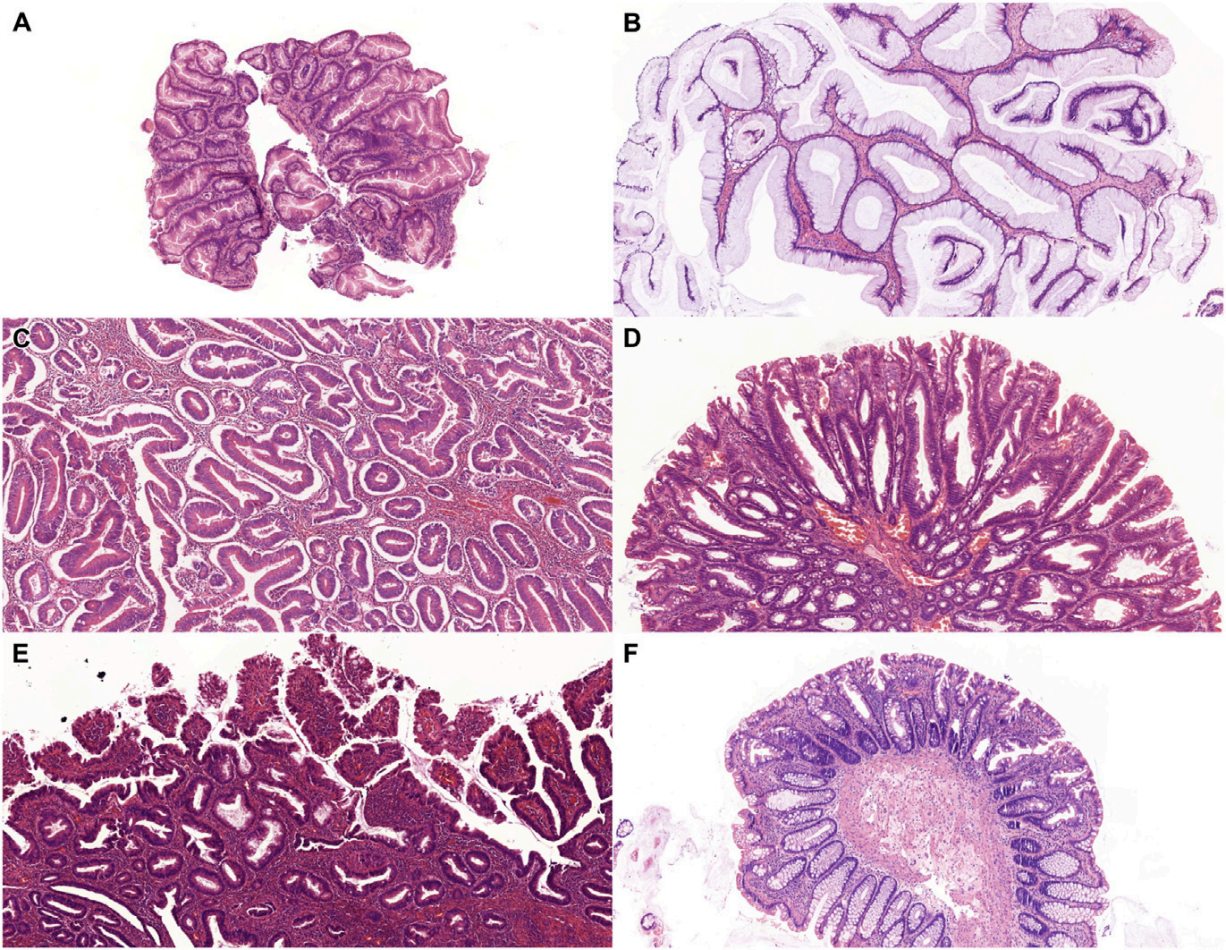
Microscopic features of IBD-associated, non-conventional dysplasias. **(A)**: Serrated lesion, NOS (HE, 5x). **(B)**: Hypermucinous dysplasia (HE, 5x). **(C)**: Goblet cell deficient dysplasia (HE, 5x). **(D)**: Sessile serrated lesion-like dysplasia (HE, 5x). **(E)**: Traditional serrated adenoma-like dysplasia (HE, 5x). **(F)**: Serrated epithelial change (HE, 5x). Abbreviations: HE - Hematoxylin and eosin, IBD - Inflammatory bowel disease, NOS - Not otherwise specified.

Serrated NOS dysplasia proved to be the most frequently observed subtype of NCD (n = 6; 30%), followed by hypermucinous (n = 4; 20%) GCD (n = 4; 20%) SSL-like (n = 2; 10%), and TSA-like dysplasia (n = 1; 5%). The clinicopathological features of the identified NCDs are summarized in Table 2. Serrated epithelial changes (SEC) were identified in 3 cases (15%). Since SEC is not unequivocally classified as dysplasia in current guidelines, these lesions are not included in the table. Figure 2 represents the identified histological subtypes.

**TABLE 2:** Clinicopathological characteristics of the identified NCDs.

	Serrated lesion, NOS (n = 6)	Hypermucinous dysplasia (n = 4)	GCD (n = 4)	SSL-like dysplasia (n = 2)	TSA-like dysplasia (n = 1)
Age at diagnosis (years)	Mean: 64.8 Median: 66 (56–74)	Mean: 65 Median: 66 (48–83)	Mean: 56.8 Median: 55 (50–72)	Mean: 51.5	70
Male-to-female ratio	3:3	3:1	1:3	2:0	1:0
Type of IBD (n)	UC: 5 CD: 1	UC: 4 CD: 0	UC: 3 CD: 1	UC: 1 CD: 1	UC: 1 CD: 0
Localization of IBD (n)	Left colon: 5 Right colon: 1 Pancolitis: 0	Left colon: 0 Right colon: 0 Pancolitis: 4	Left colon: 2 Right colon: 1 Pancolitis: 1	Left colon: 1 Right colon: 0 Pancolitis: 1	Left colon: 0 Right colon: 0 Pancolitis: 1
Duration of IBD (years)	Mean: 15.6 Median: 15 (4–21)	Mean: 15.9 Median: 15 (9–28)	Mean: 16.6 Median: 16 (8–26)	Mean: 20	Mean: 9
Localization of dysplasia (n)	Left colon: 5 Right colon: 1	Left colon: 4 Right colon: 0	Left colon: 2 Right colon: 2	Left colon: 2 Right colon: 0	Left colon: 1 Right colon: 0
Grade of dysplasia (n)	Low-grade: 5 High-grade: 1	Low-grade: 4 High-grade: 0	Low-grade: 4 High-grade: 0	Low-grade: 2 High-grade: 0	Low-grade: 1 High-grade: 0
Size of dysplasia (cm)	Mean: 0.59 Median: 0.45 (0.3–2.4)	Mean: 0.55 Median: 0.4 (0.5–1.6)	Mean: 0.73 Median: 0.5 (0.3–1.4)	Mean: 0.65	Mean: 0.40
Endoscopic appearance of dysplasia (n)	Polypoid: 6 Flat: 1	Polypoid: 2 Flat: 2	Polypoid: 1 Flat: 3	Polypoid: 2 Flat: 0	Polypoid: 0 Flat: 1
Association with conventional dysplasia (n)	4	3	3	1	1
Association with other NCDs (n)	0	2	3	1	1
Association with adenocarcinoma (n)	4	2	4	1	0

Abbreviations: CD, Crohn’s disease; GCD, goblet cell deficient; NCD, non-conventional dysplasia; NOS, not otherwise specified; SSL, sessile serrated lesion; TSA, traditional serrated adenoma; UC, ulcerative colitis.

A significant association was observed between the development of NCD and co-occurrence (*P < 0.001*), localization (*P = 0.001*), size (*P = 0.002*), macroscopic appearance (*P = 0.01*), grade (*P = 0.005*), histological subtype (*P = 0.003*), pT stage (*P = 0.003*), pM stage (*P = 0.047*) of colorectal carcinoma and microsatellite status (*P < 0.001*). However, no significant associations were found between NCDs and patient age (*P = 0.396*), gender (*P = 0.968*), type (*P = 0.319*), duration (*P = 0.481*), and extent of IBD (*P = 0.415*), macroscopic appearance (*P = 0.145*), and grade of NCD (*P = 0.865*), pN stage of invasive carcinoma (*P = 0.039*), or the presence of lymphovascular invasion (*P = 0.119*).

### Evaluation of IBD-associated adenocarcinomas in patients with NCD

Out of the 20 patients with NCD, 12 (60%) developed colorectal adenocarcinoma during follow-up. Altogether 75% of tumors were located in the left colon (n = 9). The detected adenocarcinomas were predominantly classified as low-grade (n = 11; 91.7%). Histologically, the majority of these cancers were conventional adenocarcinomas (n = 8; 66.7%), followed by mucinous (n = 2; 16.7%), GCD (n = 1; 8.3%), and signet ring cell carcinomas (n = 1; 8.3%). The average tumor size was 3.64 cm (median: 3.5; range: 0.2–7.5). Macroscopic examinations most frequently revealed a polypoid morphology (n = 7; 58.3%), followed by sessile (n = 3; 25%) and flat lesions (n = 2; 16.7%). Tumors were identified as T1 (n = 2; 16.7%), T2 (n = 3; 35%), T3 (n = 4; 33.3%), and T4 (n = 3; 25%), respectively. Nodal involvement was observed only in N1 and N2 stages (n = 1; 8.3% and n = 2; 16.7%). Distant metastases were detected in 4 cases (33.3%). Immunohistochemical analysis of microsatellite status was performed in 14 cases. Microsatellite instability was identified in 1 case (8.3%), while 7 cases (58.3%) reflected mismatch repair proficiency. In the remaining 4 cases, no immunohistochemical or molecular genetic testing was performed.

### Clinicopathologic characteristics of IBD-associated, combined conventional dysplasia and NCD

Conventional dysplasia and NCD combined were identified in 15 cases (25.3%). The mean age of patients was 61.2 years (median: 58; range: 46–83). Male predominance was observed (n = 9; 75%), and most patients were treated with UC (n = 11; 73.3%). Patients were diagnosed with dysplasia 14.5 years after IBD diagnosis (median: 16; range: 5–28 years). UC affected the left colon in 8 cases (53.3%), and dysplasia was localized to the left colon in the majority of cases (n = 12; 80%). The average size of dysplasia proved to be 0.86 cm (median: 0.6; range: 0.2–2.5). In 8 cases (53.3%), the dysplasia appeared as polypoid during the endoscopic examination, while it remained flat in the rest of the cases (n = 7; 46.7%).

A significant association was found between the co-presence of conventional dysplasias and NCDs and dysplasia size (*P = 0.045*), development (*P = 0.025*), localization (*P = 0.032*), macroscopic appearance (*P = 0.047*), multifocality (*P = 0.017*), histological subtype of colorectal carcinoma (*P = 0.033*), pT (*P = 0.014*), pN stage (*P = 0.012*), and microsatellite status (*P = 0.002*). There was no significant association observed with patient’s age (*P = 0.37*), gender (*P = 0.684*), subtype (*P = 0.735*), duration (*P = 0.158*) and extent of IBD (*P = 0.468*), localization (*P = 0.19*), grade (*P = 0.224*), and macroscopic appearance of dysplasia (*P = 0.286*), size (*P = 0.056*) and grade (*P = 0.052*) of colorectal carcinoma, pM stage (*P = 0.089*), and the presence of lymphovascular invasion (*P = 0.057*).

### Evaluation of IBD-associated adenocarcinomas in patients with combined conventional dysplasias and NCD

Nine (15.8%) patients who acquired both conventional and NCDs were diagnosed with colorectal carcinoma during follow-up. The average age of patients at neoplasia diagnosis proved to be 61.6 years (median: 56; range: 46–83). The male-to-female ratio was 6:3. In accordance with the above-detailed results, most patients (n = 5; 55.6%) were diagnosed with UC, and IBD extent was observed either in the left colon (n = 5; 55.6%), or as pancolitis (n = 3; 33.3%). On average, patients were treated with IBD for 15.3 years before any kind of dysplasia diagnosis (median: 15.5; range: 5–26). The carcinoma was mostly localized to the left colon (n = 6; 66.6%), with average size of 3.7 cm (median: 3.8; range: 0.2–7.5). During the grossing examination, the majority of carcinomas were observed infiltrative (n = 6; 66.6%) and were identified histologically as low-grade (n = 8; 88.9%). Multiplicity was observed in 5 cases (55.6%) during follow-up. The most common histological subtype proved to be conventional adenocarcinoma (n = 5; 55.6%), followed by mucinous (n = 2; 22.2%), signet ring cell (n = 1; 11.1%), and GCD (n = 1; 11.1%) patterns. These carcinomas were mostly identified in an advanced, pT3 or pT4 stage (n = 7; 77.8%), with lymph node and distant metastasis observed in 3-3 cases, respectively (33.3%; 33.3%). Microsatellite instability was identified in a single case (11.1%).

### Survival analysis

**FIGURE 3:**
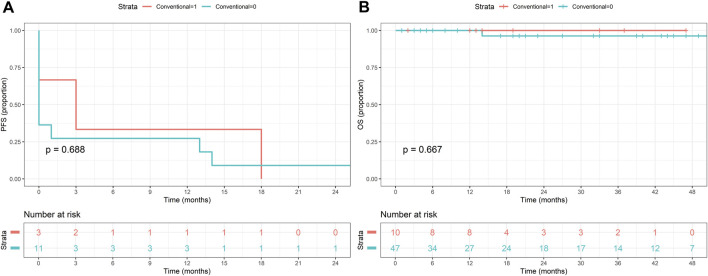
Kaplan-Meier estimates of progression-free **(A)** and overall survival **(B)** in patients with conventional dysplasia.

**FIGURE 4:**
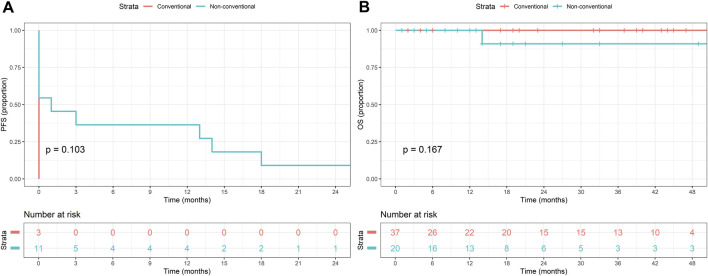
Kaplan-Meier estimates of progression-free **(A)** and overall survival **(B)** in patients with non-conventional dysplasia.

**FIGURE 5:**
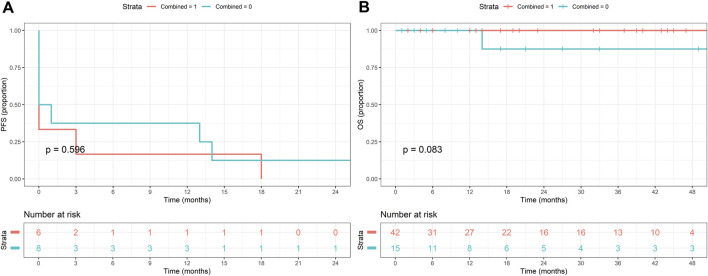
Kaplan-Meier estimates of progression-free **(A)** and overall survival **(B)** in patients with combined conventional and non-conventional dysplasia.

Kaplan-Meier analysis was performed to determine survival estimates (Figures 3–5). In patients with conventional dysplasia, there was no significant association found either in PFS (*P = 0.688*) or in OS (*P = 0.667*). Similar results were observed in patients with NCD in PFS (*P = 0.103*) and OS (*P = 0.167*), and in combined cases (*P = 0.596* in PFS, and *P = 0.083* in OS), respectively. The reason for that may lie in the fact that the cases comprising this study were procured in a period when NCDs were not yet discovered, therefore, random biopsy sampling was not yet widely applied. The other possible explanation may be the relatively short period of follow-up, therefore, the authors not only plan to expand the database but plan to reperform the survival analysis in 5 and 10 years, as well.

## Discussion

Choi et al introduced a new classification of IBD-associated dysplasias, namely, NCDs [5]. The presence of NCDs is currently considered to predict a poorer prognosis, as they are more frequently associated with aneuploidy, high-grade dysplasias, and high-risk carcinomas. Some subtypes may appear flat during the endoscopic examination, making their detection challenging. Given the limited information about these lesions, their clinical and histological identification remains difficult.

In our study, we reviewed the neoplastic specimens of IBD patients with histological samples from the University of Szeged (Hungary) between 2011 and 2015 to identify NCDs, to perform statistical analysis of clinicopathological factors potentially influencing prognosis, and to compare them with conventional dysplasias. During the study period, 57 patients were included in our database, with a mean age of 63.7 years (median: 65, range: 33–94), and male predominance (n = 34; 59.6%). The majority of patients were treated for UC (n = 41; 71.9%).

Conventional dysplasias were observed in 47 patients. The average age proved to be 65.2 years (median: 66; range: 33–94), and male predominance was observed in this group, as well (n = 31; 65.9%). Patients were mostly diagnosed with UC (n = 33; 70.2%), with left colon localization (n = 22; 46.8%). The average IBD duration until dysplasia diagnosis was 14.8 years (median: 14; range: 1–37). In accordance with IBD localization, dysplastic lesions were mainly identified in the left colon (n = 31; 65.9%). Altogether 12 patients (25.5%) were diagnosed with colorectal adenocarcinoma during follow-up, that were mainly localized to the left colon (n = 8; 66.7%). The majority were identified as low-grade (n = 10; 83.3%). In half of the cases (n = 6), multiplicity was observed, as well. Seven cases (58.3%) were characterized as conventional adenocarcinoma, followed by mucinous (n = 3; 25%), signet ring cell (n = 1; 8.3%), and GCD (n = 1; 8.3%) patterns. Most cases (n = 8; 66.7%) were found in an advanced, T3 or T4 stage. A significant association was found between the presence of conventional dysplasias and dysplasia localization, size, endoscopic appearance, grade, macroscopic appearance of colorectal carcinoma, and pT stage.

In our cohort, NCDs were identified in 20 patients (35.1%), predominantly around the 6th decade of life, with a slight male predominance (n = 12; 60%), associated with UC (n = 14; 70%). Of note, UC, NCD subtypes, as well as colorectal adenocarcinomas, were predominantly localized to the left colon in this cohort. During revisional histological examination, serrated NOS dysplasia was detected in 6 cases (30%), followed by hypermucinous (n = 4; 20%) GCD (n = 4; 20%), SSL-like (n = 2; 10%) and TSA-like dysplasia (n = 1; 5%). Out of the 20 cases, a total of 12 (60%) also developed colorectal adenocarcinoma, which was found to be predominantly low-grade (n = 11; 91.7%) and conventional adenocarcinoma subtype (n = 8; 66.7%). Rarer tissue subtypes were also identified, however, as the association of non-conventional carcinoma subtypes with NCDs is not fully understood, further conclusions on this issue cannot be made. The majority of these adenocarcinomas were detected at advanced T3 and T4 stages (together n = 7; 58.3%), and 4 cases (33.3%) were diagnosed with the M1 stage. A significant correlation was observed between NCDs and colorectal carcinoma development, localization, tumor size, macroscopic appearance, grade, histological subtype, pT and pM stag, and microsatellite status.

Conventional dysplasias and NCDs were present combined in 15 cases (25.3%). In accordance with the above-mentioned data, the mean age of patients was 61.2 years (median: 58; range: 46–83), male predominance was observed (n = 9; 75%), and most patients were treated with UC (n = 11; 73.3%). Patients were diagnosed with dysplasia 14.5 years after IBD diagnosis (median: 16; range: 5–28 years). Altogether 9 patients (15.8%) were diagnosed with colorectal carcinoma, as well, that were identified as low-grade (n = 8; 88.9%), and in 5 cases (55.6%) as multiple. Several histological subtypes were identified, including conventional adenocarcinoma (n = 5; 55.6%), mucinous (n = 2; 22.2%), signet ring cell (n = 1; 11.1%), and GCD adenocarcinoma (n = 1; 11.1%). A significant association was found between the co-presence of conventional dysplasias and NCDs and dysplasia size, development, localization, macroscopic appearance, multifocality, histological subtype of colorectal carcinoma, pT, pN stage, and microsatellite status.

International findings and our work suggest that the detection of IBD-associated NCD may be a key issue in future gastrointestinal pathology, as it was found to be common in neoplastic specimens in our consecutive retrospective study (20/57; 35.1%). It is also important to emphasize that, based on the work of Choi et al [5]. using North American data, several NCD subtypes are flat, making them difficult or impossible to detect endoscopically. Among the patients involved in our study, the majority of NCDs were polypoid (n = 11; 55%); however, it must be noted that at the time of sampling NCD subtypes had not yet been described, so the samplers may not have been aware of the significance of potentially flat lesions. Furthermore, in our data, serrated NOS dysplasia was found to be the most common, whereas the CCD and DPD subtypes observed in the US population were not identified at all. The different proportions of histological subtypes could account for the differences in endoscopic morphology. Based on our findings, the presence of NCD predisposes the development of colorectal adenocarcinoma and predicts a poorer prognosis, as it is associated with tumors of more advanced stages. Moreover, these tumors were predominantly found to be microsatellite stable, rendering them ineligible for treatment with Programmed Cell Death Ligand 1 inhibitors according to current guidelines [9].

Conventional dysplasias have already been widely investigated by many study groups during the last 4 decades, therefore, all the above-mentioned clinicopathological factors have been identified. On the other hand, NCDs have been identified in recent years, therefore, they have only been in the focus of attention for a short period, resulting in limited information. It must be emphasized that such consecutive, retrospective, cohort study has not been performed before in a Central European population. Furthermore, our study aimed to investigate cases with the co-occurrence of conventional dysplasias and NCDs, representing results as the first of their kind.

With the emergence of novel morphological variants, the histopathological characteristics are not fully described, making their diagnosis particularly challenging. This complexity is compounded by the requirement of the confirmation of at least two gastrointestinal pathologists to diagnose IBD-associated neoplasms, as stipulated by current international guidelines [10]. However, non-primary healthcare institutions may lack the necessary specialization, as well as access to advanced diagnostic tools such as immunohistochemistry and molecular genetic testing. Furthermore, the poor prognosis associated with IBD-related NCDs may be attributed to their higher rates of aneuploidy and their frequent association with high-risk carcinomas. Given these challenges, some experts advocate for randomized biopsy sampling and more rigorous patient monitoring in cases of non-conventional dysplasia [11].

In conclusion, IBD-associated NCDs require increased attention during histopathological evaluation. Larger cohort studies from Hungary and the Central European region are needed to gain a more comprehensive understanding of their clinical behavior. Based on current knowledge, patients may benefit from close follow-up involving endoscopic sampling, randomized biopsies, and, in cases where NCD is diagnosed, complete removal of the lesion is recommended [11].

## Data Availability

The original contributions presented in the study are included in the article/supplementary material, further inquiries can be directed to the corresponding author.
